# Glances at the Hospitals

**Published:** 1900-07-28

**Authors:** 


### GLANCES AT THE HOSPITALS.

### THE STAMFORD INFIRMARY.

The receipts amounted to £2,233, and the expenditure to £2,654. An adverse balance of £471 exists. The total number of in-patients was 353, divisible into 296 general cases and 57 infectious. The average stay in the infirmary was a little over 26 days, and the daily average number resident was 20. Including out-patients, 96 were under treatment at the beginning of the year; 530 were admitted during the year; 561 were discharged in one way or another; and 65 were still under treatment. An analysis shows that 244 were cured; 106 relieved; 134 did not attend or left; 5 were deemed incurable; and 20 died. In the isolation wards a total of 57 patients were treated. Of these, 35 recovered; 3 died; and 19 remained under treatment. Of the 57 cases, 39 were scarlet fever, 10 were typhoid, and 8 diphtheria. Anæsthetics—the favourite being the A. C. E. Mixture—were administered 147 times.

### THE YORK COUNTY HOSPITAL.

This institution issues its one hundred and fifty-ninth annual report. During 1899 the receipts were: Ordinary income, £5,425; legacies, £28; transferred from capital account for improvements, £364; capital items, £17,119. The expenditure was: House and other expenses, £6,093; exceptional expenditure, £236; capital items, £17,119, making a total of £23,449, and leaving a balance of £2,453 due to the treasurer. The committee say they view with much concern the fact that for the last three years the income has been insufficient to enable them to discharge their liabilities; and yet we may remark that the workmen's and miscellaneous collections have increased, and the average cost per bed occupied has been reduced from £57 10s. to £56 19s. Altogether the admissions reached 1,054, and of these 390 were medical cases, 574 were surgical, and 90 were ophthalmic. Of the medical cases 326 were discharged cured or relieved, of the surgical cases 526, and of the ophthalmic 87. There was a total of 92 deaths, and 116 patients remained under treatment at the end of the year. The statistics of the out-patients' department show that 277 remained under treatment at the beginning of the year, and that there were 6,726 new patients. Of general diseases there were 58 treated, and 2 deaths; of diseases of the respiratory system, 77 cases, with 11 deaths; of nervous diseases, 70 cases, with 15 deaths; of urinary diseases, 19 cases, with 1 death; of alimentary cases, 86 cases, with 3

deaths; of the circulatory system, 51 cases, with 6 deaths; of various specific and infectious diseases, 56 cases, with 8 deaths. There were 10 cases of poisoning without a death. The surgical tables are also very carefully prepared, and can be followed at a glance. Twelve cases of hernia were operated on without a death. Anæsthetics were given in 499 cases; in 164 of these chloroform was administered, and in 130 ether was the agent, the others were the A.C.E. mixture alone or with ether, and nitrous oxide. The secretary is Mr. Neden, and the lady superintendent is Miss Gwyn.

